# Health of refugees settled in Australia over time and generations: a transformative mixed methods study protocol

**DOI:** 10.1136/bmjopen-2023-083454

**Published:** 2024-09-20

**Authors:** Angela J Dawson, Anita E Heywood, Sally Nathan, Abela Mahimbo, Andre MN Renzaho, Adele Murdolo, Melissa Kang, Mitchell Smith, Andrew Hayen

**Affiliations:** 1 School of Public Health, Faculty of Health, University of Technology Sydney, Sydney, New South Wales, Australia; 2 School of Population Health, Faculty of Medicine and Health, University of New South Wales, Sydney, New South Wales, Australia; 3 Dean's Unit—School of Medicine, Western Sydney University, Penrith, New South Wales, Australia; 4 Multicultural Centre for Women's Health, Melbourne, Victoria, Australia; 5 General Practice Clinical School, The University of Sydney, Sydney Medical School, Sydney, New South Wales, Australia; 6 Refugee Health, New South Wales Ministry of Health, St Leonards, New South Wales, Australia

**Keywords:** Community-Based Participatory Research, Health Services, Mortality, Health Equity, Health Services Accessibility

## Abstract

**Abstract:**

**Background:**

Refugees resettled in Australia may experience significant physical, mental and emotional health issues on arrival and difficulty accessing mainstream healthcare that often demands specialised services. It is not known if and how refugee health needs and service use change over time and generations, how this compares with the broader Australian population and what level of resourcing is required to maintain specialised services. There is also a significant knowledge gap concerning the resources and skills of refugees that can be harnessed to sustain the health and well-being of individuals and communities. Such knowledge gaps impede the ability of the health system to deliver responsive, efficient, acceptable and cost-effective care and services and limit the engagement of refugees in the coproduction of these services.

**Methods:**

This study will be the first to provide comprehensive, longitudinal, population-based evidence of refugee health, service use and the accumulated resources or assets related to positive health and well-being (compared with data on deficits, illness and death) across the lifespan and generations. This will enable a comprehensive understanding of the relationships among assets, health status, service gaps and behaviours. We will identify the assets contributing to increased capacities to protect and promote health. This evidence is essential for planning health prevention programmes.

This project has three phases: (1) employ national linked datasets to examine the health and social outcomes of refugees in Australia; (2) engage with refugees in a participatory manner to map the social, economic, organisational, physical and cultural assets in their communities and deliver an integrated model of health; and (3) codesign a roadmap of agreed actions required to attain health and well-being in communities and indicators to assess outcomes.

**Ethics and dissemination:**

Ethics and procedures—phase I:

Ethical approval for phase I was gained from the Australian Bureau of Statistics (ABS) for Person Level Integrated Data Asset microdata (unit record data) via the ABS DataLab and the NSW Population and Health Services Research Ethics Committee (2023ETH01728), which can provide a single review of multijurisdictional data linkage research projects under the National Mutual Acceptance Scheme. This will facilitate approval for the Victorian and ACT datasets. The ABS will be the integrating/linkage authority. The Centre for Health Record Linkage (CHeReL) and the Victorian Data Linkage Unit will prepare a data extract representing all data records from the dataset to provide to the ABS for linkage.

Ethics and procedures—phases 2 and 3:

Written consent will be obtained from all participants, as well as consent to publish. We have obtained ethical approval from the University of Technology Sydney Medical Research Ethics Committee; however, as we deepen our consultation with community members and receive input from expert stakeholders, we will likely seek amendments to hone the survey and World Café questions. We will also need to provide flexible offerings that may extend to individual interviews and online interactions.

**Discussion:**

This innovative approach will empower refugees and put them at the centre of their health and decision-making.

**STRENGTHS AND LIMITATIONS OF THIS STUDY**

- In phase I, it is not possible, for ethical reasons, to create an entire cohort of the Australian population to compare refugees to non-refugees. However, Australian national data will form a comparison.
- We will use multiple datasets to compare health service use against health outcomes.
- For phases II and III, the focus on key communities in urban New South Wales, Victoria and the Australian Capital Territory may mean that our data do not reflect the full experience of all refugees in Australia and their families. However, the online nature of the survey will help to engage those of refugee backgrounds from other states and territories and rural and possibly remote areas.
- The online delivery of the nominal group process may restrict participation from those who are not computer literate or do not have access to the internet; however, we will work to ensure access to those identified by both panels as critical to the process.

## Background

In the last 10 years, wars, civil conflicts and natural disasters have led to the displacement of 71 million people, the most significant number in any decade since World War II. The 2030 Sustainable Development Goal Agenda recognises the positive contributions of refugees for inclusive growth and sustainable development.^1^ Countries striving to address health inequities are looking for translatable approaches to address these refugees’ typically marginalised and often educationally disadvantaged needs to maximise their health and well-being and, therefore, contribute to their new countries. However, data on health and service needs, values and preferences for care is critical to this. A recent WHO report has identified an absence of comparable data within and across countries and over time on refugee and migrant health and the lack of disaggregation according to migratory status within global health datasets.^2^ Evidence is, therefore, critical to ensure responsive health systems and supportive environments to address the health and well-being of refugees.

Since 1991, almost 420 000 humanitarian entrants have resettled in Australia.^3^ On arrival, refugees frequently present with physical health issues, including chronic hepatitis B, latent tuberculosis infection,^4^ poorly managed chronic diseases such as diabetes^5^ and are under-immunised.^6^ Poorer perinatal outcomes have been observed among refugees compared with Australian-born women, which can have lasting effects on infants.^7^ Health issues affecting migrants may also affect refugees, such as cancer incidence that is increasing among immigrants compared with Australian-born populations, with higher rates of liver and oesophageal cancers.^8^ Refugees experience poor mental health, including conditions such as traumatic stress disorder and psychological distress at rates higher than other Australians, related to both the premigration and postmigration experience.^9^

Poor mental and physical health can be exacerbated by acculturation stress, social isolation and limited access to and use of health services due to language barriers, low health literacy, past health-seeking beliefs and behaviours and a lack of health professional cultural competence.^10^ Refugees have been reported to have higher use of general practitioner services^11^ and emergency departments for acute presentations^12^ than others in the community, although evidence is mixed.^13^ While the health of newly arrived refugees is likely to be poor, there is a lack of research examining a range of health issues over time beyond initial screening. There is only one Australian study indicating an ongoing high risk of poor mental health specific to post-traumatic stress syndrome (PTSD) on arrival among parents.^14^ Longitudinal evidence is available in other countries, such as Sweden, that showed lower rates of substance use disorders in refugees over time that converged with the Swedish-born rate over time.^15^ There are no population-based studies in Australia using data linkage that can provide long-term insights into the physical and mental health of refugees.

Population-based studies on subgroups such as refugees require unique identifiers in datasets and registers. These identifiers are not readily available for refugees in the existing datasets.^16^ For example, while the Australian Immunisation Register contains Medicare identifiers, it lacks information on refugee status, preventing measuring coverage rates at the practice or health district level for efficient planning and service delivery for these populations.^6^ Datasets such as the *45 and Up* cohort study represent older Australians fluent in English, while the Household, Income and Labour Dynamics in Australia survey and the Longitudinal *Study* of Australian Children include few refugees, with migrants comprising less than 15% of the sample. These challenges have meant that low priority has been given to identifying refugee-background populations in Australian datasets over time and generations.^17^

The Building a New Life in Australia (BNLA) dataset is the only longitudinal study gathering data, but it is only from a sample of refugees who arrived in Australia between May and December 2013.^18^ The BNLA collects information about housing, English language proficiency, employment, financial circumstances, immigration and trauma experience and self-reported mental and general health. The small sample, participant dropout and between-arrival mean long-term health outcomes cannot be examined, and generalisability is limited. This dataset also focuses on mental health, excluding a comprehensive assessment of physical health.

No research has examined national measures of health service utilisation, such as Medical Benefits Scheme (MBS) or Pharmaceutical Benefits Scheme (PBS) data, to provide information concerning the services and medicines refugees use during their postsettlement period and as indicators of continuity of care over time. Study populations in available research are heterogeneous, combining culturally and linguistically diverse (CALD), migrant and refugee groups^13^ and are limited by small samples of ethnically diverse groups who may have different needs and resources^19^ with a focus on arrival screening, including, immunisation that varies across states and territories.^20^ Other research focuses on the refugee experience of health services shortly after arrival, highlighting problems with access to care and a focus on health deficits, illness and death.

Efforts to partner with communities to improve refugee health in Australia have also been impacted by a lack of population-level data. Multicultural health services in states and territories in Australia work have little specific data about refugee healthcare needs, preferences and experiences from which to plan and develop services for this population over time. Meaningful partnerships with refugee communities are required to proactively codesign culturally competent health services,^21^ based on what is working and ensure the promotion of healthier living and environments.^22^ Ensuring consumer and community involvement and engagement as equal partners in processes to improve health services is not only important to refugees^13^ but also core to state government efforts, including the New South Wales (NSW) Ministry of Health.^23^ This approach puts refugees at the centre of their health decision-making, emphasising fairness and commitment to address the social determinants of health.^24^

The research gaps, therefore, highlight a lack of data and a lack of focus on positive health and well-being, as well as strengths and assets among refugees and their communities that can be harnessed for health education and prevention. Few studies examine the inter-relationships between health, social connections, work and education from the perspectives of refugees, a perspective that can contribute to improving health service access and health-promoting behaviours at the individual, family, community and organisational level over time and across generations.^25 26^ This emphasises the need for a health assets approach that can support healthy practices^27^ to determine current and future health potential.^28^ Health assets to improve public health are well explored in groups such as adolescents but have been largely ignored in refugees, except for specific studies in the USA.^29^,^31^ The analysis of assets actively engages and empowers individuals and communities to define local issues and coproduce local solutions to planning health services.

This paper presents the protocol for research that will build a comprehensive national picture of the health status of resettled refugees in Australia, their healthcare service use and health-promoting or protective factors that can proactively inform service delivery and cocreate solutions for policy and practice across generations.

## Methods

### The study aims, design and setting

This multiphase mixed methods study will provide individual, community and population-based data about refugee health and consists of three distinct phases (table 1). In phase I, we will conduct a novel data linkage study using existing datasets to deliver large-scale insights into the health of refugee populations in Australia and detailed data on various refugee groups according to language, religion, culture, age, gender, country of birth etc that will provide evidence with a high level of external validity and applicability for policymaking.^32^ In phase II, we will conduct mixed methods research to provide a complementary picture of refugee health that focuses on the accumulated resources or assets related to promoting and protecting health across the individual, family and community life span. The third phase will involve the dissemination and discussion of the synthesised results from phases I and II and extensive consultation to codesign an evidence-based plan for refugee health and well-being.

**Table 1:** The aims and design of each study phase

Phase I	Retrospective cohort study using linked data.
Aim	Describe the hospital-based healthcare, medication use and morbidity and mortality outcomes of refugees and project the future health service needs of resettled refugees and their children in Australia.
Phase II	Mixed methods health asset mapping.
Aim	Map the sociodemographic characteristics and health-promoting and protective factors of refugees at individual, organisational and community levels in different waves of arrival since 1991 and subsequent generations to determine opportunities to develop individual supports and flourishing communities.
Phase III	Qualitative participatory action study.
Aim	Cocreate a roadmap for planning refugee healthcare services and health promotion in a partnership approach with communities and service providers in New South Wales and Victoria.

This research will focus on refugees in NSW, the Australian Capital Territory (ACT) and Victoria (Vic). NSW and Vic are the two Australian states with the highest proportion of refugees resettled since 2001, when detailed visa information became available (418 716 total, 37% or 155 536 in NSW, 134 319 or 32% in Vic).^3^ The data linkage study will explore the health status and service use of all refugees and their children in the jurisdictions of NSW, ACT and Vic. We will engage with at least 15 refugee communities that reflect the main ethnic and cultural groups who have arrived in Australia since 2001 across key local government areas in Sydney and Melbourne (settlement locations of the largest number of refugees), where we will undertake recruitment in phases II and III. These communities include Karen, Chin, Assyrian, Chaldean, Kurdish, Persian, Hazara, Tadjik, Pashtun, Ethiopian, Oromo, Tigrinya, Dinka, Somali, Congolese, Tamil and Lhotshampas. Data will be collected from September 2024 to the end of 2027.

### Methodological approaches and conceptual frameworks

**Figure 1:**
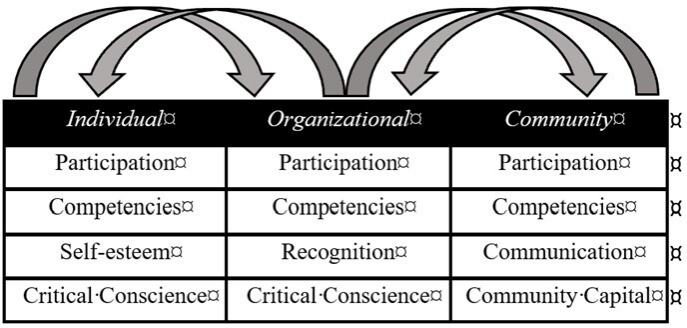
Model of empowerment.^47^

We will employ transformative mixed methods that are aligned with a social justice paradigm that acknowledges research as not merely an objective investigation but as an activity that has the power to influence and transform individuals, communities and systems.^33^ Our study recognises the sociopolitical inequities of historically marginalised populations and will be conducted following best-established practices that include collaborating with refugees, ensuring refugee communities are engaged in decision-making and supporting their agency in effecting change. These best practice principles transverse the preinvestigation to postinvestigation phases and are sensitive to the different origins, experiences and sociodemographic backgrounds of refugees and their families. Strategies such as involving community members in pilot tools to provide feedback on sensitive questions and implementation strategies will be critical to this research.^34^ In addition, researchers will engage in critical self-reflexivity that recognises the role of the researcher’s positionality and values with the aim of addressing social inequalities and injustices through the research process, findings and recommendations.^35^ Therefore, rigour will be demonstrated by ensuring that data are collected and analysed with the involvement of refugees and used to facilitate social change. Collaboration with refugee community members will be achieved through governance structures detailed below and participatory methods in phases II and III to identify assets, needs and solutions for health enhancement. A model of empowerment will underpin the research and the outcomes sought (see figure 1). This model has four related dimensions within individual, organisational and community empowerment that have been described as pathways to health.^36^ These dimensions will inform our research tools and analysis in phases II and III.

This model will be strengthened with the addition of the theory of capitals acknowledging the interconnected resources that enable communities to achieve social equity and well-being^28^ and the related theory of salutogenesis,^37^ focusing attention on factors that support human health and well-being, not factors that cause disease or pathogenesis. This strength-based theory seeks to understand the foundations of positive patterns of health and contributing resources or assets. Morgan’s Assets Model for Public Health^38^ will be used to link the evidence identified in phase II to the action required in phase III to attain and evaluate approaches to enable individuals, organisations and communities to develop and sustain positive health (figure 1).

### Patient and public involvement

This study will involve refugees and engage them in the research. We will follow the Guidance for Reporting Involvement of Patients and the Public Checklist^39^ to report our research findings, which will ensure that we describe all the stages and ways that refugees are involved in the research. A refugee review panel^40^ will be established before the study begins to advise on community engagement strategies and provide input into research questions and emerging findings. This panel will also monitor the study’s compliance with cultural safety principles and guide the dissemination of the results. Throughout the study, we will present our methods and emerging findings and invite members to provide input. We plan to provide training in research methods for members who are interested and seek doctoral students of refugee backgrounds to work with us. In phase I, the panel will assist with decision-making concerning the variables to examine in the data linkage study, and in phase II, the approach to the exploration of health assets and recruitment of participants. The panel will also contribute to the interpretation of findings including the synthesis of data in phase III and the consensus-building process to identify key priorities for health services and programming. Refugee leaders and representatives from relevant communities across key states will be invited to participate on the panel. Meetings will be held in hybrid mode to maximise participation and technical support provided.

### Research and health service engagement and involvement

A knowledge translation panel comprised of senior decision-makers in research and health services will facilitate engagement with policymakers to ensure policy-relevant outcomes and dissemination. This will include health professionals engaged in service provision across diverse areas such as maternal child health, mental health, cancer, disability and infectious disease, managers of government and not-for-profit programmes and networks, alliances, peak bodies, and professional associations.

### Phase I: Retrospective cohort study, using linked data

#### Aim

Describe the hospital-based healthcare, medication use and morbidity and mortality outcomes of those granted visas under the Australian Humanitarian Program since 2001 (when visa information became available) and project the future health service needs of resettled refugees and their children.

#### Participant selection and recruitment

All humanitarian entrants, identified by age at arrival and country of birth, will be included. In the 10 years until June 2023, the countries of birth with the largest number of arrivals in Australia were Iraq, the Syrian African Republic, Afghanistan, Myanmar, Iran, The Democratic Republic of Congo and Pakistan (200–204, 851 and 866)^41^

##### Cohort and size of the population/s

We expect approximately 300 000 people to be in the entire cohort. The cohort will be defined as:

- All humanitarian entrants to Australia identified by visa type (subclasses 200–2017, 785, 798, 815–818, 850, 851, 866).
- Children of those defined above born in NSW, Vic or the ACT.

Refugee populations will be identified from the Australian Federal Government Department of Home Affairs’ Migration Database. Populations will be identified through data linkage to births data to the registrar of births, deaths and marriages in NSW, the ACT and Vic.

### Data collection

We will use linked data of approximately 300 000 refugees from The Person Level Integrated Data Asset (PLIDA) managed by the Australian Bureau of Statistics (ABS) to examine health and social outcomes across Commonwealth agencies, including ABS, Australian Taxation Office, Department of Education, Department of Health and Aged Care, Department of Social Services and Department of Home Affairs. National datasets of refugee entrants will be linked with state-based datasets from NSW and Vic, where most refugees reside. The ACT will also be included. Children of refugee parents will be identified through birth registrations and linked to their mother or father.

### Data analyses

#### Key variables of interest and statistical methods

We will use indirect standardisation by age and sex to calculate expected rates for the refugee population from the overall Australian reference population for outcomes such as mortality, hospitalisations for mental health conditions and cancer incidence, among others. We will estimate standardised incidence (or mortality) ratios with corresponding 95% CIs. We will also compare the incidence of health outcomes in refugees against other immigrants from the source countries. Our focus and analysis will be on the following key priority areas in table 2. We will use Poisson (or negative binomial regression) to obtain rate ratios for comparing rates across refugee groups—such as by country of origin—provided there are sufficient numbers among subgroups. In cases where specific strata are too small, we will consider combining them into meaningful composite groups or conducting pooled analyses. This approach allows us to retain the ability to compare across different origins while ensuring that group sizes remain adequate for robust descriptive analysis.

**Table 2:** Data areas of focus

Priority area	Details
Mortality	We will estimate rates of death by cause (eg, heart disease, cancer, suicide) among refugees using ABS death data. We will also compare rates across refugee groups (eg, country of origin) using methods for analysing count data (eg, Poisson regression) that will allow adjustment for potential confounding (such as for age).
Hospitalisation and ED	Rates of hospitalisation for chronic disease (eg, diabetes, heart disease and mental health) and emergency department presentations by syndrome. We will use similar statistical methods to analyse mortality data.
PBS and MBS	We will estimate prescription rates for treatments such as those for mental health issues, such as anxiety and depression, using PBS data. Correspondingly, we will determine how many refugees were covered by mental healthcare plans from MBS data.
Notifiable conditions	We will obtain rates of diagnoses of notifiable conditions, such as chlamydia, gonorrhoea and HIV. Statistical methods will be as indicated above.
Cancer	We will estimate the incidence of cancer in the refugee population
Child development perinatal	We will use the Australian Early Childhood Development Census to compare refugee children with others on key measures of child development. We will use this to describe maternal and child health.

ABSAustralian Bureau of StatisticsEDemergency departmentMBSMedical Benefits SchemePBSPharmaceutical Benefits Scheme

### Outcome

Data visualisation describing patterns in morbidity, mortality and health service use across generations will be made publicly available using open-source software such as Paraview.

### Phase II: Mixed methods health asset mapping, a survey and participatory methods

#### Aim

Map the sociodemographic characteristics and health-promoting and protective factors among refugees at individual, organisational and community levels in different waves of arrival since 1991 (oldest available data)^3^ and subsequent generations to determine opportunities to develop resilient, flourishing communities.

##### Cross-sectional survey

This survey will map individual assets and examine the association with positive outcomes. We hypothesise that individual health asset scores (social participation, health literacy, acculturation, critical consciousness) will be associated with self-reported positive physical and mental health.

### Participant selection and recruitment

We will select 15 refugee communities by ethnicity from local government areas in NSW, ACT and VIC. Maximum variation sampling^42^ will gather a sample that is as diverse as possible to capture the range of individuals in specific communities (by gender, age, etc). A matrix will be used for selection to capture geographical location, population size, cultural and socioeconomic diversity and timing of resettlement. Eligible criteria will be refugees and their children (>16 years for consent purposes). We will work with national, state and local community groups, as guided by our refugee review panel and culturally informed recruitment principles,^43^ to provide information about the study and build trust and rapport to obtain the required sample size and gather data.^44^ With the assistance of our research and stakeholder partners, we will engage community liaison officers (CLOs) who speak the language of each community and are known to the community to assist with recruitment.

Participants will be recruited online and via respondent-driven sampling (RDS), a network sampling technique useful for reaching large numbers of refugees.^45^ A small, purposeful sample of the target population (seeds) will be asked to complete the survey. Individuals who have an extensive network or a high reputation in the community will be invited to be seeds by the CLOs. Seeds will invite contacts who are also members of the target population by using recruitment coupons. Participants will receive a gift voucher for completing the survey and for successfully recruiting other respondents in additional waves. The total sample size for the RDS is estimated to be n=400, which is based on estimating dichotomous characteristics with a CI precision of ±3.5%, and a design effect of 2. Previous work^46^ indicates that it will be feasible to recruit this sample size with 3 seeds within each of the 20 communities and 3 gift vouchers per participant. Flyers will be posted on message boards and on the front desks of relevant services. Online recruitment will also take place through (1) posting and sharing on Facebook and Twitter (X) through relevant social networks, such as refugee organisations and collectives and (2) advertising on partner organisations social media pages.

### Data collection

We will use an online survey (table 3) to assess each domain of individual empowerment, as specified in figure 1, ^47^. Study instruments have been validated for use with CALD populations, including refugees, in multiple languages. The survey, study information and informed consent form will be provided online using the University of Technology Sydney (UTS) licence of Qualtrics in 16 local languages and English. Gender, age, country of birth, visa subclass and year of arrival data and employment status data will also be collected. CLOs will share the survey via email, WhatsApp and other messaging tools, and potential respondents can scan the QR code on the message or via the study flyer. Hard copies will also be available for those who do not wish to complete it online. CLOs can assist participants with low literacy in completing the survey on their phones or personal devices. The survey will take, on average, 40 min to complete, after which participants will be invited to the community assets mapping and, if interested, provide contact details.

**Table 3:** Study instruments comprising the survey and the health assets they measure by domain

Domain	Instrument	Description
Participation	Social Participation Scale	8-item scale assessing involvement in social activities.^60^
Competencies	Immigrant Settlement Services Literacy Scale	30 questions, 3 categories on migrant’s ability to access services, question providers and input into service design.^61^
	Vancouver Acculturation Index	20 questions assess migrants’ orientations towards mainstream and heritage traditions.^62^
Self-esteem	Rosenberg Self-Esteem Scale	10-item scale that measures global self-worth.^63^
Critical consciousness	Critical Consciousness Scale	22-item measure comprised of three subscales: perceived inequality, egalitarianism and sociopolitical participation.^64^
Health	12-item Short Form Survey (SF-12)	12 measure self-report physical and mental well-being.^65^

### Data analysis

Descriptive statistics will examine the proportion of assets in each domain and distribution according to each self-reported health indicator. Linear and logistic regressions will determine whether each asset moderates association with self-reported physical and mental health.

### Outcomes

A database of individual assets and their association with self-reported health outcomes and sociodemographic characteristics and data visualisation to explore these patterns.

### Participatory research using World Cafés

Organisational and community asset mapping will be employed to identify social, economic, organisational, physical and cultural assets in refugee communities and how this capacity can be assessed and harnessed for health.

#### Participant selection and recruitment

We will select individuals from the 15 communities for collective assets mapping workshops, including those who participated in the individual assets mapping and who self-nominated to be involved (selection described above). Recruitment will be further enhanced by flyers, radio and website advertisements, in addition to identifying interested people from the survey (as above). CLOs will provide information sessions on the World Café method to all potential participants and select up to 30 people that reflect the diversity of genders, ages, employment, education, year of arrival and disability in each community and gain consent.

#### Data collection

We will employ a group-based innovative qualitative method known as World Cafés to provide structured knowledge sharing between community members.^48^ CLO’s in each community will coordinate a 3-hour World Café at an appropriate time and venue. Groups of six will participate in 20 min conversational rounds facilitated by a nominated group member before moving to another table. Each table will discuss the following topics: *our affiliations, our talents and skills, and measuring our capacity*. These topics are based on a framework for mapping capacity described by Jackson *et al*^49^ that is aligned to the organisational and community dimensions of the empowerment model to be used in this study (figure 1). Group work will be recorded at each table by a nominated note keeper on butcher’s paper and emerging themes distilled by the group in a large, facilitated session at the end of the workshop. The Refugee Advisory Committee (RAC) will provide input into the analysis as part of a triangulation strategy^50^ to integrate data.

##### Data analysis

A thematic analysis, guided by an a priori framework of asset topics, of the notes and recordings from the World Cafés discussions will be undertaken in two stages: (1) within each community and (2) across communities, with the latter serving as the basis for the development of indicators of community capacity. Data will be coded using the software package NVivo. Themes will be discussed among researchers and the RAC to achieve inter-rater reliability and consensus reached on coding.

### Integration of phases I and II data

This stage involves developing a comprehensive view of refugee health. The results from phase I (quantitative linked data) will be integrated with phase II (quantitative survey and qualitative assets mapping) into coherent and meaningful ‘meta-inferences’.^51^ The meta-inference phase aims to determine what assets can be harnessed for resilient and flourishing communities based on identified needs. A triangulation protocol^50^ will be used by CIs to converge the data using a matrix^52^ to understand the relationships between assets, health and behaviours. Attention will be paid to social factors and assets (ie, gender) and systemic/structural inequities. We will identify patterns where assets increase capacities and promote health and compare this within and across communities. The matrix will be reviewed with the RAC.

#### Outcome

‘Meta-themes’ will be identified for use in phase three and linked to form a cohesive whole resulting in a visual understanding of refugee health that identifies health status, needs, social determinants of refugee health across communities, waves of arrival and between generations and the individual organisation and community assets available and required to improve health and well-being.

### Phase 3: participatory planning using an online national nominal group (NG) process

**Figure 2:**
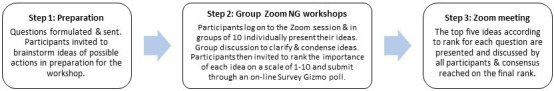
The nominal group process. NG, nominal group.

This phase aims to cocreate a roadmap for planning refugee healthcare services and promotion in partnership with communities and service providers in NSW, ACT and Vic. A 2-hour online national NG process will be held to codesign a roadmap and recommendations for improving health for refugee communities and to inform health promotion, service policy and planning (see figure 2). The NG process has been successfully employed in refugee communities to increase cancer screening and treatment.^53^ The meta-themes developed in phase II will direct questions for participants to respond to and anonymously rank to achieve consensus and establish priorities. This process will identify actions to improve health, implementation processes and indicators to assess outcomes.

#### Participants

Based on the advice of our refugee review panel, CLOs and knowledge translations panel, we will approach community leaders, policymakers and peak bodies seeking interest in participation. We anticipate 30 participants. A week before conducting the NG sessions, participants will be emailed an explanatory statement outlining the aims and the structure of the NG session, a consent form for signing, and the list of questions to be asked throughout the session (see step 1, figure 2).

#### Data collection

Participants will rank their responses to the questions during the online session (see step 2, figure 2) using a 9-point scale that favours consensus.^54^

#### Data analysis

Data will be analysed according to NG standard scoring procedures.^55^ Ratings of 1–3 indicate low importance, 4–6 indicate moderate and 7–9 show the highest degrees of importance. A decision will be made on the top five responses to questions at a Zoom meeting (step 3, figure 2). We will hold a national workshop to finalise the process of prioritisation and consensus-making with a broader group of stakeholders.

#### Outcomes

The first national codesigned roadmap document outlining objectives and milestones for refugee health promotion programmes and service delivery will be developed and disseminated.

### Ethics and dissemination

#### Ethics and procedures for phase I

Ethical approval for phase I was gained from the ABS for PLIDA microdata (unit record data) via the ABS DataLab and the NSW Population and Health Services Research Ethics Committee (2023ETH01728), which can provide a single review of multijurisdictional data linkage research projects under the National Mutual Acceptance Scheme. This will facilitate approval for the Victorian and ACT datasets. The ABS will be the integrating/linkage authority. CHeReL and the Victorian Data Linkage Unit will prepare a data extract representing all data records from the dataset to provide to the ABS for linkage.

We will produce aggregated tables, figures and regression models. In all outputs, we will consider privacy issues. In reports of our analyses, we will use the guidelines suggested by the NSW Ministry of Health in our reporting, as well as meet the requirements of each data custodian. Specifically, we will take steps to ensure that we use disclosure control methods to avoid disclosure of information (such as identity disclosure, attribute disclosure and inferential disclosure). Methods that we will adopt include cell suppression, restructuring tables or figures to mitigate risks against disclosure and restricting reporting for populations with small denominators.^56^

#### Ethics and procedures: phases II and III

Written consent will be obtained from all participants, as well as consent to publish. We have obtained ethical approval from the UTS Medical Research Ethics Committee; however, as we deepen our consultation with community members and receive input from expert stakeholders, we are likely to seek amendments to hone the survey and World Café questions. We will also need to provide flexible offerings that may extend to individual interviews and online interactions.

## Discussion

Since the end of World War II, nearly a million refugees^57^ have made their home in Australia. However, we know very little about their health and well-being after arrival and if this differs from the broader population. Our innovative study will, for the first time, identify the health needs and assets of refugees by synthesising evidence from multiple sources at the population, community and individual levels. The result will be a codesigned framework for planning refugee health promotion and delivery of healthcare services in Australia with a focus on NSW, ACT and Vic. This will be the first time that data has been harnessed internationally alongside the voices of members of cultural community groups to understand refugee health needs and address social determinants. This research will incorporate the traditional epidemiological model of measuring health status and service utilisation with an ‘assets’ model that identifies capability and opportunities to activate solutions to health. This approach will enable the delivery of the world-first consensus roadmap to direct the creation of culturally safe and sustainable health services and health-promoting environments with refugee communities.

### Strengths and limitations of this study

In phase I, it is not possible, for ethical reasons, to create a full cohort of the Australian population to compare refugees to non-refugees. However, Australian national data will form a comparison. While many of the datasets in our analysis depend on people accessing health services (eg, the PBS and MBS), other datasets (eg, deaths and the Australian Early Childhood Development Census) do not. Additionally, data linkage in Australia has a very high rate of linkage^58^ and previous research has found high linkage rates even for marginalised communities.^59^ Additionally, we will use multiple datasets to compare health service use against health outcomes.

Phases IIand III have several limitations. The focus on key communities in urban NSW, Vic and the ACT may mean that our data do not reflect the full experience of all refugees in Australia and their families. However, the online nature of the survey will help to engage those of refugee backgrounds from other states and territories and rural and possibly remote areas. The online delivery of the NG process may restrict participation from those who are not computer literate or do not have access to the internet; however, we will work to ensure access to those identified by both panels as critical to the process.
