# Supplementary material for: Health of refugees settled in Australia over time and generations: a transformative mixed methods study protocol
Source: BMJ Open. 2024 Sep 20;14(9):e083454. doi: 10.1136/bmjopen-2023-083454 (PMC11418575; doi:10.1136/bmjopen-2023-083454)
Supplement: Supplementary file 1 [file bmjopen-2023-083454.reviewer_comments.pdf]

PEER REVIEW HISTORY

BMJ Open publishes all reviews undertaken for accepted manuscripts. Reviewers are asked to complete a checklist review form and are provided with free text boxes to elaborate on their assessment. These free text comments are reproduced below.

ARTICLE DETAILS

**TITLE (PROVISIONAL)**	The health of refugees settled in Australia over time and generations: A transformative mixed-methods study protocol.
**AUTHORS**	Dawson, Angela; Heywood, Anita; Nathan, Sally; Mahimbo, Abela; Renzaho, Andre; Murdolo, Adele; Kang, Melissa; Smith, Mitchell; Hayen, Andrew

VERSION 1 - REVIEW

**REVIEWER NAME**	Razum, Oliver
**REVIEWER AFFILIATION**	Bielefeld University, Epidemiology and International Public Health
**REVIEWER CONFLICT OF INTEREST**	None declared
**DATE REVIEW RETURNED**	11-Mar-2024

**GENERAL COMMENTS**	Thank you for your description of this important study.<br><br>Mandatory revisions:<br><br>Has funding been secured for all elements of the planned study with the 2021 “Ideas” grant? On what date has data collection started, which data have already been collected, and how much time will be available for the study?<br><br><br>Technical issues:<br><br>P. 4, Line 28: post, not poster<br><br>The quantitative part of the Methods section is not yet well developed:<br>- P. 6, lines 5-7: You will have troves of data, but what exactly are you going to do with them? Without hypotheses, you may find random associations. (This question is partly answered on p. 8/9). Moreover, once you stratify e. g. by origin, group sizes will be small. Maybe provide an example of a power calculation?<br>- P. 6, lines 41/42: proportion of what? Please report denominator as well as absolute numbers.<br>- P. 6/7: How will you involve refugees in deciding which quantitative analyses to conduct?<br><br>P. 14, lines 39 f.: This should have been said at the beginning of the text.<br><br><br>Out of interest: Do your quantitative data provide any information on exposures throughout the life course, e. g. having been detained in one of Australia’s extraterritorial “processing facilities” before coming

	to mainland Australia? (Admittedly, this particular exposure applies only to a small number of persons).

**REVIEWER NAME**	Quayyum , Zahidul
**REVIEWER AFFILIATION**	BRAC University, BRAC James P Grant School of Public Health
**REVIEWER CONFLICT OF INTEREST**	None
**DATE REVIEW RETURNED**	12-Apr-2024

**GENERAL COMMENTS**	This paper is a protocol paper and is based on a comprehensive conceptual research framework. The researcher has explained that they will employ transformative mixed methods and aligning it with a social justice paradigm and acknowledging that their research will not merely for investigation, but the process or activity is expected to have the power to influence and transform individuals, communities, and systems. It is understood that the study recognizes the socio-political inequities of historically marginalized populations and that the authors will conduct the study following best-established practice that will ensure collaborating with refugees, ensuring refugee communities are engaged in decision making and supporting their agency in effecting change. However, it would be useful if the best - established practices explained a bit here in the manuscript so the readers understand the contexts of different origins of refugees who are in living different context of social capital.<br>The researcher has suggested that for their Phase 3, (Participatory planning using an online national nominal group process) they will use a two-hour online national nominal group (NG) process that will be held to co-design a roadmap and recommendations for improving health for refugee communities and to inform health promotion, service policy and planning. However, is it possible to make use of the national conferences to such national group to conduct this co-design or co-creation activities and achieve consensus and establish priorities.

VERSION 1 – AUTHOR RESPONSE

REVIEWER 1 COMMENT

Has funding been secured for all elements of the planned study with the 2021 “Ideas” grant? On what date has data collection started, which data have already been collected, and how much time will be available for the study?

RESPONSE

This is a four-year study, and funding has been secured for all aspects of the study as described in the protocol. We have delayed the start of the project due to the time required to secure ethics and expect to receive the linked data, and we will be embarking on the collection of data outlined in phase two in the second half of this year. We have identified the funding in the declarations section at the end of the manuscript.

REVIEWER 1 COMMENT

Technical issues:

P. 4, Line 28: post, not poster

RESPONSE

Corrected

REVIEWER 1 COMMENT

The quantitative part of the Methods section is not yet well developed:

- P. 6, lines 5-7: You will have troves of data, but what exactly are you going to do with them? Without hypotheses, you may find random associations. (This question is partly answered on p. 8/9).

Moreover, once you stratify e.g. by origin, group sizes will be small. Maybe provide an example of a power calculation.

RESPONSE

We have combined our responses to the two comments.

We appreciate the reviewer's concern regarding the potential issue of small group sizes and low statistical power when stratifying by country of origin in our study of refugees. We have added further detail on page 8 and 9 regarding our approach.

We address this concern by highlighting several points that support our approach:

1. Large Sample Size of Administrative Data: Our study uses a comprehensive administrative dataset that includes a substantial number of refugee records. This large sample size allows us to mitigate the issue of small group sizes even after stratifying by origin. We believe that the richness of this dataset provides a solid foundation for meaningful descriptive analysis across various subgroups. However, we will ensure that we do not disclose health information (e.g., by not reporting cells in tables with small sample sizes, e.g. fewer than five).

2. Focus on Descriptive Analyses: It is important to note that our study primarily focuses on descriptive analyses. The main objective is to summarise and describe the characteristics and distributions of different refugee groups rather than to conduct inferential analyses. Descriptive statistics are well-suited to provide valuable insights even when group sizes are relatively small.

3. Importance of Stratification: Stratifying by country of origin is crucial for uncovering differences in demographic, social, and economic characteristics that are specific to various refugee groups, particularly given that the countries of origin have changed over time. These insights are essential for informing targeted interventions and policies tailored to the unique needs of different origins. The benefits of understanding these nuanced differences outweigh the potential drawbacks of smaller group sizes in descriptive studies.

4. Composite or Pooled Analyses: In cases where specific strata are too small, we consider combining them into meaningful composite groups or conducting pooled analyses. This approach allows us to retain the ability to compare across different origins while ensuring that group sizes remain adequate for robust descriptive analysis.

5. Effect Size and ‘Clinical Significance’: Our descriptive analysis also considers the magnitude and practical significance of observed differences. Even with smaller groups, identifying substantial and clinically meaningful variations provides valuable insights that can guide policy and practice for refugee support and integration.

6. Ethical and Equity Considerations: Stratifying by country of origin ensures that the unique experiences and outcomes of different refugee groups are recognised and addressed. This approach promotes inclusivity and equity in our research, aligning with ethical standards and the overarching goal of understanding and supporting diverse refugee populations.

7. Indirect standardisation: we will use indirect standardisation. Noting that the standard error for estimates of the standardised incidence or mortality ratio are dependent on the total number of observations (rather than the number of age and sex-specific observations), this approach is more robust for comparing incidence rates in smaller populations to, say, national incidence rates.

We hope these points clarify our rationale and support the robustness of our approach. We believe that the insights gained from stratifying by country of origin are vital for informing effective and equitable refugee policies and interventions.

REVIEWER 1 COMMENT

- P. 6, lines 41/42: proportion of what? Please report the denominator as well as absolute numbers.

RESPONSE

We have clarified the following:

NSW and Vic are the two Australian states with the highest proportion of refugees resettled since 2001, when detailed visa information became available (418,716 total, 37% or 155,536 in NSW, 134,319 or 32% in Vic.)

REVIEWER 1 COMMENT

- P. 6/7: How will you involve refugees in deciding which quantitative analyses to conduct?

RESPONSE

We will present both our quantitative and qualitative methods findings to both the refugee and translation panels and will be available to answer questions and receive feedback at these meetings and outside them. While the refugee panel includes members with health, medical and research qualifications, not all will have the expertise to contribute to the data analysis across all phases of the study. However, we welcome panel members who are interested in making suggestions and those with statistical skills to participate in the analysis where appropriate. We are also planning to provide the refugee panel with training in areas that they identify need. This can include an introduction to quantitative research methods. We have clarified this on p. 7 where have outlined how people of refugee background will be involved in this study:

Throughout the study, we will present our methods and emerging findings and invite members to provide input. We plan to provide training in research methods for members who are interested and seek doctoral students of refugee backgrounds to work with the research team.

REVIEWER 1 COMMENT

P. 14, lines 39 f.: This should have been said at the beginning of the text.

RESPONSE

This paragraph has now been moved to the start of the background.

REVIEWER 1 COMMENT

Out of interest: Do your quantitative data provide any information on exposures throughout the life course, e. g. having been detained in one of Australia's extraterritorial "processing facilities" before coming to mainland Australia? (Admittedly, this particular exposure applies only to a small number of persons).

RESPONSE

The data linkage study will only provide information concerning health on arrival in Australia. Qualitative data may provide some insights pertaining to pre-arrival experiences.

REVIEWER 2 COMMENT

However, it would be useful if the best -established practices explained a bit here in the manuscript so the readers understand the contexts of different origins of refugees who are in living different context of social capital.

RESPONSE

We believe the reviewer is referring to this sentence "Our study recognises the socio-political inequities of historically marginalised populations and will be conducted following best-established practice that includes collaborating with refugees, ensuring refugee communities are engaged in decision making and supporting their agency in effecting change". In response, we have added the following:

These best practice principles transverse the pre- to post-investigation phases and are sensitive to the different origins, experiences and socio-demographic backgrounds of refugees and their families. Strategies such as involving community members in pilot tools to provide feedback on sensitive questions and implementation strategies will be critical to this research [1].

REVIEWER 2 COMMENT

The researcher has suggested that for their Phase 3, (Participatory planning using an online national nominal group process) they will use a two-hour online national nominal group (NG) process that will be held to co-design a roadmap and recommendations for improving health for refugee communities and to inform health promotion, service policy and planning. However, is it possible to make use of the national conferences to such national group to conduct this co-design or co-creation activities and achieve consensus and establish priorities.

RESPONSE

We have clarified this process with the following sentence in the NG process section

We will hold a national workshop to finalise the process of prioritisation and consensus-making with a broader group of stakeholders.
